# Blood biomarker dynamics in people with relapsing multiple sclerosis treated with cladribine tablets: results of the 2-year MAGNIFY-MS study

**DOI:** 10.3389/fimmu.2025.1512189

**Published:** 2025-02-03

**Authors:** Heinz Wiendl, Frederik Barkhof, Xavier Montalban, Anat Achiron, Tobias Derfuss, Andrew Chan, Suzanne Hodgkinson, Alexandre Prat, Letizia Leocani, Klaus Schmierer, Finn Sellebjerg, Patrick Vermersch, Hulin Jin, Anita Chudecka, Andreas Kloetgen, Dongdong Lin, Lidia Gardner, Nicola De Stefano

**Affiliations:** ^1^ Department of Neurology, Institute of Translational Neurology, University of Münster, Münster, Germany; ^2^ Department of Radiology and Nuclear Medicine, Amsterdam UMC, Vrije Universiteit, Amsterdam, Netherlands; ^3^ Queen Square Institute of Neurology and Centre for Medical Image Computing, University College London, London, United Kingdom; ^4^ Department of Neurology-Neuroimmunology, Centre d’Esclerosi Múltiple de Catalunya (Cemcat), Hospital Universitario Vall d’Hebron, Barcelona, Spain; ^5^ Multiple Sclerosis Center, Sheba Academic Medical Center, Ramat Gan, Israel; ^6^ Faculty of Medicine, Tel-Aviv University, Tel-Aviv, Israel; ^7^ Department of Neurology, University Hospital Basel, Basel, Switzerland; ^8^ Department of Neurology, Inselspital, Bern University Hospital, University of Bern, Bern, Switzerland; ^9^ Ingham Institute for Applied Medical Research, University of New South Wales Medicine and Liverpool Hospital, Sydney, NSW, Australia; ^10^ Department of Neurosciences, Université de Montréal, Montréal, QC, Canada; ^11^ Department of Neurology, University Vita-Salute San Raffaele, Milan, Italy; ^12^ Experimental Neurophysiology Unit, Scientific Institute IRCCS San Raffaele, Milan, Italy; ^13^ Department of Neurorehabilitation Science, Casa di Cura Igea, Milan, Italy; ^14^ The Blizard Institute, Centre for Neuroscience, Surgery and Trauma, Barts and The London School of Medicine and Dentistry, Queen Mary University of London, London, United Kingdom; ^15^ Clinical Board Medicine (Neuroscience), The Royal London Hospital, Barts Health NHS, Trust, London, United Kingdom; ^16^ Danish MS Center, Department of Neurology, Copenhagen University Hospital - Rigshospitalet, Glostrup, Denmark; ^17^ Department of Clinical Medicine, University of Copenhagen, Copenhagen, Denmark; ^18^ Univ. Lille, Inserm U1172 LilNCog, CHU Lille, FHU Precise, Lille, France; ^19^ Clinical Measurement Sciences, Merck Healthcare KGaA, Darmstadt, Germany; ^20^ Clinical Research Services, Cytel Inc., Geneva, Switzerland; ^21^ Clinical Measurement Sciences, EMD Serono Research & Development Institute, Inc., an affiliate of Merck KGaA, Billerica, MA, United States; ^22^ Neurology & Immunology Medical Unit, EMD Serono Research & Development Institute, Inc., an affiliate of Merck KGaA, Billerica, MA, United States; ^23^ Department of Medicine, Surgery and Neuroscience, University of Siena, Siena, Italy

**Keywords:** multiple sclerosis, cladribine tablets, biomarkers, transcriptomics, immunophenotyping, immune reconstitution therapy

## Abstract

**Background and objectives:**

Cladribine tablets (CladT) represent an effective immune reconstitution therapy, administered in short treatment courses over two consecutive years. To better understand the amplitude of immune changes, we performed a comprehensive analysis during the 2-year study period for the entire MAGNIFY-MS population (N=270). In addition to lymphocyte kinetics, we studied intracellular cytokines serum proteins, and their associations with clinical outcomes. To put these changes into perspective, we analyzed transcriptional changes in T and B cells and associated biological pathways before and after each treatment course with CladT.

**Methods:**

Immunophenotyping and transcriptomics were performed at regular visits with major differences reported between baseline (BL) and after each yearly treatment course. Assessments included: lymphocyte dynamics, RNA sequencing (B and T cells), intracellular cytokines, serum proteins (immunoglobulins [IgG and IgM], and serum neurofilament light chain [sNfL]). Clinical measures included: MRI activity, annualized relapse rate (ARR), 6-month confirmed disability progression (6mCDP), timed 25-foot walk (T25FW), and 9-hole peg test (9HPT).

**Results:**

All B, T and NK cells were reduced at month (M)3 after CladT administration, except regulatory B cells which increased above BL from M3 to M24. Naïve and transitional B cells recovered at M6; all other B and T cell subsets remained below BL levels. Reductions in all NK cell subtypes were observed at M3, CD16^low^CD56^bright^ and NKp46 cells reconstituted at M6 and M12 respectively. Changes in genes and pathways associated with innate and adaptive immune response were observed after CladT treatment, along with reductions in pro-inflammatory cytokine-producing B and T cells and increases in anti-inflammatory cytokine-producing T cells. IgG and IgM levels remained above the lower limits of normal in most participants. sNfL levels decreased, remaining reduced by M24. Significant reductions in the annualized combined unique active lesion count occurred from M2 onwards. ARR was 0.11 (95% confidence interval: 0.09,0.15), with 83% participants free of qualifying relapses. Over 90% of participants were free of 6mCDP, around 87% had no confirmed progression on T25FW and 9HPT. No significant correlations were seen between clinical parameters and lymphocyte dynamics to M6. The safety profile was consistent with previous reports.

**Discussion:**

Deep longitudinal immunophenotyping, analysis of transcriptional changes, reduction in cells expressing pro-inflammatory cytokines, along with the marker of neuroaxonal damage provide novel and innovative evidence of CladT rebalancing the immune system towards a more homeostatic and less pathogenic state.

**Clinical Trial Registration:**

https://clinicaltrials.gov/study/, identifier NCT03364036.

## Introduction

Multiple sclerosis (MS) is a chronic immune-mediated disease that affects the central nervous system (CNS). Most approved disease-modifying therapies (DMTs) require continuous administration and act by suppressing the immune system to deliver beneficial effects, whereas cladribine tablets 10 mg (CladT) control the disease by delivering an immune reconstitution effect (1–8).

Understanding patterns of depletion and repopulation, following such immune reconstitution therapy (IRT) in the context of global clinical trials, aids our understanding of CladT’s effect on immunity and MS pathophysiology. Other DMTs, such as anti-CD20s, do have long-lasting effects but this is often associated with chronic depletion of immune cells and continuous immunosuppression (9), whereas IRT offers sustained efficacy with preserved immunocompetence for infections and vaccine response (10). Deep immunophenotyping can provide important insights on CladT’s unique mechanism of MS disease control. In recent years Epstein-Barr virus (EBV, which causes life-long latent infection of memory B [B_mem_] cells) has been associated with MS etiology (11, 12). As such, using treatments that provide a lasting effect on B_mem_ cells may prove particularly efficacious. While it is known that CladT significantly depletes B_mem_ cells, its effect on other aspects of immunopathophysiology of MS is less clear (13).

The primary objective of the MAGNIFY-MS study (NCT03364036) was to determine the onset of action of CladT on neuroradiological activity in people with highly active relapsing multiple sclerosis (RMS), as was previously reported using 6-month MRI data (14). The secondary objective of MAGNIFY-MS was to characterize the pattern and kinetics of lymphocyte reduction and repopulation during treatment with CladT. We previously published 12-month data from a MAGNIFY-MS sub-study (n=57) following the first CladT treatment course (15). In this report, we describe deep immune-phenotyping after two CladT treatment courses in the entire MAGNIFY-MS population (n=270). In addition to lymphocyte kinetics, we analyzed changes in cytokine producing B and T cells from a blood biomarker sub-study, as well as RNA sequencing of CD3^+^ and CD19^+^ cells, and changes in serum neurofilament light chain (sNfL) levels. We also investigated possible associations and correlations between changes in immune cells and traditional clinical outcomes, presented to 2 years post treatment initiation for the first time (including presence of MRI lesions, qualifying relapses, 6-months confirmed disease progression [6mCDP], and progression on timed 25-foot walk [T25FW] and 9-hole peg test [9HPT]).

## Methods

### Study design and participants

MAGNIFY-MS was a 2-year, phase IV, open-label, single-arm study in which eligible participants with highly active RMS received CladT (3.5 mg/kg cumulative dose over 2 years). Participants received 2 weeks of active treatment per course (week 1 and 5 of each year), with the start of the first week of treatment in year 1 being considered baseline (BL, **Supplementary Figure 1**). The primary endpoint of this study, along with design and eligibility criteria have previously been reported (14, 15). Of particular note, participants were excluded if they had a lymphocyte count outside the normal laboratory limits, or if they had previous exposure to alemtuzumab, fingolimod, mitoxantrone, natalizumab, or ocrelizumab. Analyses for the 2-year period are described herein.

### Peripheral blood sampling

Peripheral blood mononuclear cells (PBMCs) were collected at indicated time points. Panels of B, T, and NK cells were analyzed using surface cell markers as described previously (15) at BL, month (M)3, M6, M12, M15, M18 and M24. Absolute cell counts and median percentage change from BL were assessed for cell subtypes and immunoglobulins. For the purposes of immunophenotyping, CD19^+^ B cells were analyzed as part of the TBNK cell panel, other B cell subtypes were analyzed as part of the full B-cell panel (Covance Central Laboratory Services, Inc. 8211 SciCore Drive, Indianapolis, IN 46214-2985, USA). Bulk RNA sequencing of CD19^+^ B cells and CD3^+^ T cells of PBMCs was carried out for 11 participants selected at random from the blood biomarker sub-study at BL, M3, M12, M15, and M24. Additional details are provided in **Supplementary Appendix 1**.

### Serum proteins

Serum levels of immunoglobulin (Ig)G and IgM were analyzed by nephelometric assay using reference ranges provided by Covance at BL, M3, M6, M12, and M24 as previously described (15). sNfL levels were analyzed at BL, M3, M6, M12 and M24 using Quanterix NF-light chain assay method on Quanterix Simoa HD-1 Analyzer (Labcorp/Monogram, 345 Oyster Point Blvd, South San Francisco, CA 94080, USA). Obtained sNfL values were analyzed using Wilcoxon signed rank tests comparing data at the respective visit versus BL. sNfL values were normalized using Z-scores to adjust for age and body mass index (BMI) based on a control population, using the method previously described by Benkert et al. (16) Z-score interpretation as per Benkert et al: ≤-1, reduced relative to age- and BMI-matched reference population; ~0, similar relative to age- and BMI-matched reference population, >1, elevated relative to age- and BMI-matched reference population.

### Intracellular cytokines

For participants of the blood biomarker sub-study, cytokine-producing T and B cells were analyzed at the previously described time points, with blood collected at three additional time points (M1, M2, and M14). For analysis of cytokines, 1×10^6^ of PBMC purified from whole blood was used. We studied stimulated and non-stimulated B and T-lymphocytes for individual intracellular cytokines comparing absolute values and median change from BL of each B and T cell intracellular cytokine by visit with 95% confidence intervals (CI) of the mean per stimulation method. B cell intracellular cytokines were stimulated with CpG and PMA/Iono. T cell intracellular cytokines were stimulated with Anti-CD3/CD28 or Anti-CD3/CD28 and PMA/Iono.

### Clinical measures and MRI

Clinical outcomes were assessed in all enrolled patients. Annualized relapse rate (ARR) of qualifying relapses was estimated from a Poisson regression model, adjusted for age and Expanded Disability Status Scale (EDSS) at BL (≤3, >3). EDSS, T25FW, and 9HPT scores were recorded at BL and M3, M6, M12, M15, M18, and M24. Definitions for 6mCDP, and progression on T25FW or 9HPT are provided in **Supplementary Appendix 1**. All patients were included in the assessment of EDSS progression. Patients with relapse-associated worsening were not excluded from the analysis. MRI scans were performed at screening, BL, and at M1, M2, M3, M6, M12, M15, M18, and M24 following the initiation of CladT. Initial exploratory correlation analysis included participants with both B-cell and MRI assessments up to M6 (MAGNIFY-MS study primary endpoint). Reported changes in combined unique active (CUA), T1 gadolinium-enhancing (Gd+), and active T2 lesions were correlated with B regulatory (B_reg_) and B_mem_ cell counts to investigate associations between fast CladT onset of action and lymphocyte dynamics. For measuring MRI activity, changes in CUA, T1 Gd+, and new or enlarging T2 lesion counts were compared between BL and post-BL visits using a mixed-effects linear model for repeated measures. The correlation between change in immune cell subsets and MRI from BL to M6 was assessed with Cramer’s V method (**Supplementary Appendix 1**). We also performed correlation analysis using Spearman correlation coefficient between BL and each following timepoint from M3 to M24 to guide interpretation of the results. We compared fold-changes/percent changes of B-lymphocytes with changes of CUA between BL and follow-up. Association of clinical outcomes and changes in lymphocyte ratio dynamics were carried out as exploratory. For this purpose, presence of clinical progression was compared to immune cells ratios. The full list of disability status categories and immune cell subset ratios are presented in **Supplementary Figure 2**.

### Statistics

Analyses were performed on the full analysis set (FAS), which included all eligible participants (people with highly active relapsing MS aged ≥18 years with an Expanded Disability Status Score ≤5.0) treated with at least one dose of CladT. All analyses were performed without adjustment for multiplicity. The study protocol and statistical analysis plan for MAGNIFY-MS have been published on ClinicalTrials.gov (NCT03364036) (17).

### Standard protocol approvals, registrations, and patient consents

Ethical approval for the MAGNIFY-MS study (NCT03364036) was obtained from independent ethics committees for each trial site, and the study was performed in line with the principles of the Declaration of Helsinki. All participants provided written informed consent before participation in the study. Participants who elected to participate in the blood biomarker sub-study signed additional informed consent form.

### Data availability statement

Any requests for data by qualified scientific and medical researchers for legitimate research purposes will be subject to Merck's Data Sharing Policy. All requests should be submitted in writing to Merck's data sharing portal https://www.merckgroup.com/en/research/our-approach-to-research-and-development/healthcare/clinical-trials/commitment-responsible-data-sharing.html. When Merck has a co-research, co-development, or co-marketing or co-promotion agreement, or when the product has been out-licensed, the responsibility for disclosure might be dependent on the agreement between parties. Under these circumstances, Merck will endeavor to gain agreement to share data in response to requests.

## Results

A total of 270 participants from the main MAGNIFY-MS study were analyzed in the FAS (median age 38.5 years; 66.7% female), including 57 participants enrolled in the blood biomarker sub-study (**Table 1**). Baseline characteristics for participants in the sub-study were consistent with the overall main study population.

**Table 1:** Baseline demographics and characteristics.

	Main study population (N=270)	Blood biomarker sub-study population (n=57)
Female, n (%)	180 (66.7)	35 (61.4)
Age in years, n (%)		
≤40	152 (56.3)	35 (61.4)
>40	118 (43.7)	22 (38.6)
Time since onset of MS in months, mean ± SD	84.90 ± 85.472	84.94 ± 93.385
Time since diagnosis in months, mean ± SD	60.87 ± 74.489	52.54 ± 67.413
Time since first relapse in months, mean ± SD	54.44 ± 72.583	52.63 ± 80.704
Number of relapses within 12 months prior to BL, n (%)		
0	3 (1.1)	2 (3.5)
1	102 (37.8)	15 (26.3)
2	133 (49.3)	29 (50.9)
>2	32 (11.9)	11 (19.3)
EDSS score at BL, n (%)		
≤3	204 (75.6)	42 (73.7)
>3	66 (24.4)	15 (26.3)
Median (range)	2.0 (0.0–5.0)	2.5 (0.0–5.0)
Number of previous DMTs^a^, n (%)		
0	117 (43.3)	30 (52.6)
1	88 (32.6)	13 (22.8)
2	50 (18.5)	10 (17.5)
>2	15 (5.6)	4 (7.0)
≥1 DMT within 6 months prior to start of study treatment, n (%)	123 (45.6)	–
Last DMT, n (%)		
Interferons	48 (17.8)	–
Interferon beta-1a	29 (10.7)	–
Peginterferon beta-1a	10 (3.7)	–
Interferon beta-1b	8 (3.0)	–
Interferon beta	1 (0.4)	–
Glatiramer acetate	30 (11.1)	–
Teriflunomide	23 (8.5)	–
Dimethyl fumarate	21 (7.8)	–
Immunoglobulins Nos	1 (0.4)	–
Participants with ≥1 T1 Gd+ lesion during the BL period, n (%)	136 (50.4)	23 (40.4)
Participants with ≥1 active T2 lesion (without T1 Gd+) during the BL period, n (%)	68 (25.2)	13 (22.8)
Missing, n (%)	3 (1.1)	0 (0)

a Participants were excluded from MAGNIFY-MS if they had previous exposure to alemtuzumab, fingolimod, mitoxantrone, natalizumab, or ocrelizumab.

BL, baseline; DMTs, disease-modifying therapies; EDSS, Expanded Disability Status Scale; Gd+ gadolinium-enhancing; MS, multiple sclerosis; SD, standard deviation.

### Immune cell dynamics

Quantitative immune cell changes were observed following each yearly treatment course, during study visits outlined in **Supplementary Figure 1**. All values presented are median percentage changes from BL.

**Figure 1:**
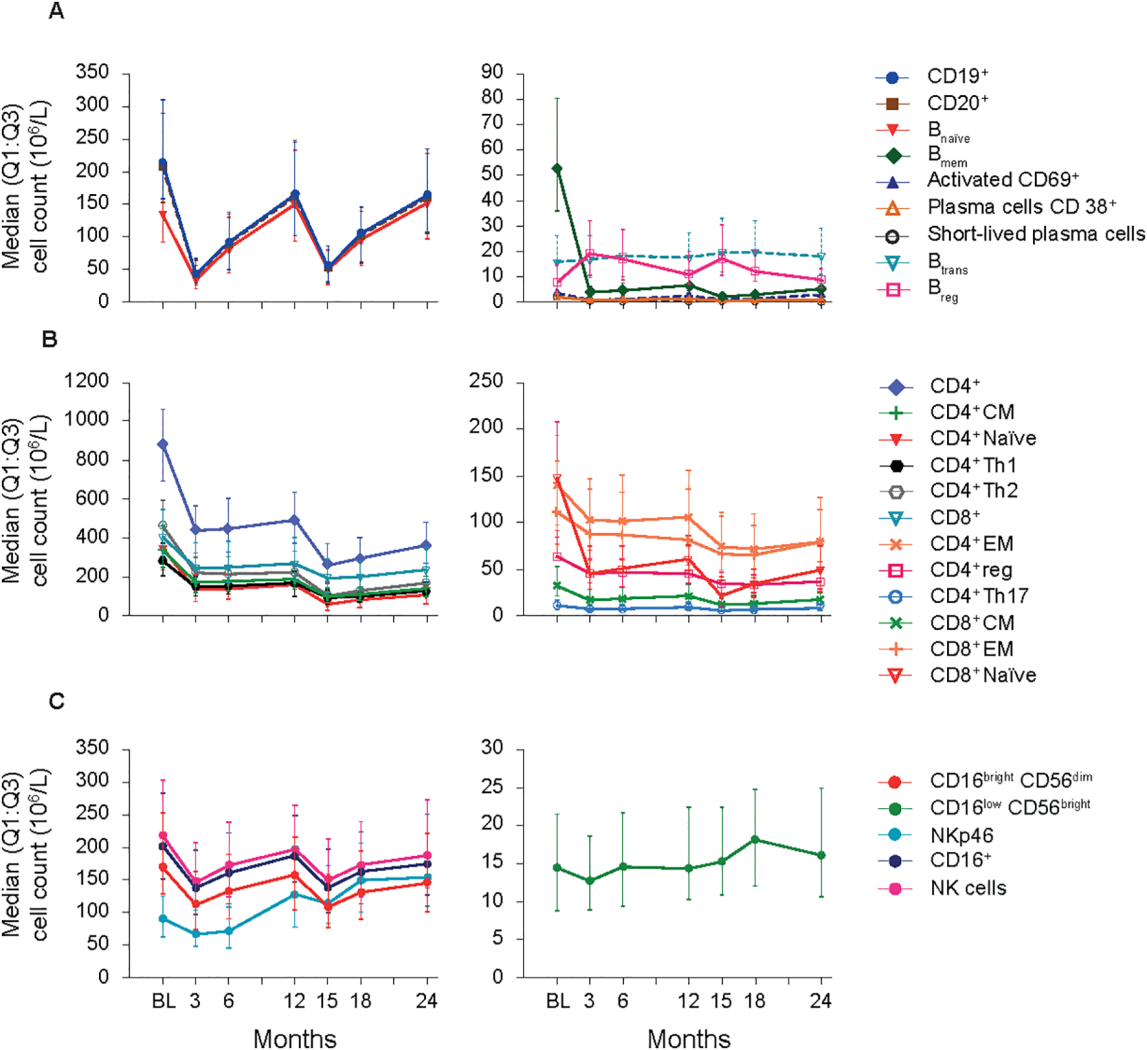
B-cell, T-cell and NK cell subsets (FAS). **(A)** B-cell panel. **(B)** T-cell panel. **(C)** NK-cell panel. BL, baseline; B_mem_, B memory; B_reg_, B regulatory; B_trans_, B transitional; CD, cluster of differentiation; CM, central memory; EM, effector memory; FAS, full analysis set; NK, natural killer; Q1:Q3, quartile range; reg, regulatory; Th, T helper cell type.

**B cell subtypes:** In the FAS, changes observed in B cell counts after the first CladT treatment were similar to those previously reported for the sub-study (15) (**Figure 1A**; **Table 2**). At the first planned post-BL visit (M3) most B cell subsets reached nadir values: B_naïve_ (-76%), CD19^+^ (-80%), CD20^+^ (-81%) and activated CD69^+^ (-74%). B_mem_, CD38^+^ plasmablasts and short-lived plasma cells reached nadir values only after the second treatment course in year 2 (M15). B_mem_ cell counts drastically reduced to -93% at M3 and to -97% at M15, this reduction was sustained throughout the observation period (M24, -89%).

**Table 2:** Median percentage change from BL for B, T and NK cell subsets and immunoglobulins (FAS).

	M3	M6	M12	M15	M18	M24
B cell subsets						
CD19^+^	-80	-61	-27	-77	-55	-28
CD20^+^	-81	-60	-25	-77	-54	-25
B_mem_	-93	-92	-87	-97	-95	-89
Activated CD69^+^	-74	-61	-29	-73	-52	-16
CD38^+^ plasmablasts	-67	-59	-55	-78	-72	-63
Short-lived plasma	-68	-57	-57	-83	-80	-70
B_naïve_	-76	-46	1.6	-69	-40	11
B_trans_	-4.1	15	12	29	11	6.3
B_reg_	111	93	31	92	34	1.6
T cell subsets						
CD4^+^	-49	-47	-40	-69	-67	-58
CD4^+^ CM	-50	-47	-40	-70	-66	-57
CD4^+^ EM	-29	-27	-20	-46	-49	-39
CD4^+^ reg	-26	-30	-26	-48	-49	-40
CD4^+^ naïve	-60	-58	-51	-84	-79	-69
CD4^+^ Th1	-44	-43	-36	-64	-63	-53
CD4^+^ Th2	-51	-52	-48	-76	-72	-63
CD4^+^ Th17	-33	-30	-18	-45	-45	-32
CD8^+^	-42	-39	-36	-57	-54	-46
CD8^+^ CM	-41	-43	-32	-63	-57	-43
CD8^+^ EM	-26	-23	-22	-44	-44	-35
CD8^+^ naïve	-68	-65	-58	-85	-78	-68
NK cell subsets						
CD16^bright^CD56^dim^	-36	-26	-11	-35	-25	-13
CD16^low^CD56^bright^	-8.9	3.7	2.6	4.8	30	17
NKp46	-21	-22	30	28	72	78
CD16^+^	-33	-22	-8.1	-29	-22	-14
NK cells	-31	-22	-7.1	-28	-18	-13
Immunoglobulins						
IgM	-7.0	-13	-20	-27	-31	-32
IgG	5.00	3.7	1.1	4.9	2.4	3.0

Light green shading signifies value above BL level; dark green shading, highest value above BL level; red shading, nadir value.

BL, baseline; B_mem_, B memory; B_naïve_, B naïve; B_reg_, B regulatory; B_trans_, B transitional; CD, cluster of differentiation; CM, central memory; EM, effector memory; FAS, full analysis set; Ig, immunoglobulin; M, month; NK, natural killer; reg, regulatory; Th, T helper cell type.

At M3, we only saw repopulation for B_reg_ cell counts (+111%), which remained elevated at M6 (+93%) and M15 (+92%). At M24, B_reg_ cell counts repopulated to around BL level (+1.6%). B_trans_ cell counts at M3 were (-4.1%), differing from the previous report. However, these cells recovered to above BL by M6 (+15%) and had the highest increase after the second treatment course (M15, +29%) and remained above BL until M24 (+6.3%). Previous sub-study observations found B_naive_ cells reaching nadir at M2 (-90%), recovering to near baseline levels at M12 (-5.0%) (15). Here, for the total study population B_naïve_ cell counts recovered after completion of each yearly treatment course (+1.6%, M12; +11%, M24, highest value).

**T cell subtypes:** Like previously published data (15), a substantial decrease in absolute values for all T cell counts was observed immediately after CladT treatment in the FAS. T cell counts decreased further after the second treatment course with nadir values at M15 for most cell subsets. CD4^+^ effector memory (EM), CD4^+^ regulatory (reg), CD4^+^ T helper (Th)17 and CD8^+^ EM reached nadir values at M18 (-49%, -49%, -45%, and -49% respectively). No T cell subtypes had recovered to BL levels at M24 (**Figure 1B**; **Table 2**). The most substantial change from BL levels was observed for CD4^+^ naïve (-84%) and CD8^+^ naïve (-85%) cells at M15. CD4^+^ Th1 and Th2 cell count reductions were like other T cell subtypes across the post-BL period, with the largest median percentage change at M15 for CD4^+^ Th1 and Th2 (-64% and -76%).

**NK cell subtypes:** Most NK cell subsets reached nadir values at M3 (**Figure 1C**; **Table 2**); however, these counts generally remained above the lower limits of normal (>100×10^6^/L) (18). The most substantial reductions were observed at M3 for CD16^bright^CD56^dim^ (-36%), CD16^+^ (-33%), CD16^low^CD56^bright^ (-8.9%), and NK (-31%) cell counts, while NKp46 cell counts were most reduced at M6 (-22%). Repopulation to above BL values was observed for CD16^low^CD56^bright^ and NKp46 cells starting at M6 and M12 respectively. At M18, CD16^low^CD56^bright^ cells increased to 30% above BL levels, differing from the previous report where CD16^low^CD56^bright^ cells remained at -9.0% at M12 (15). NKp46 cells reached BL levels at M12 and continued to increase in number after the second of two-yearly treatment courses (M24, 78%).

**Figure 2:**
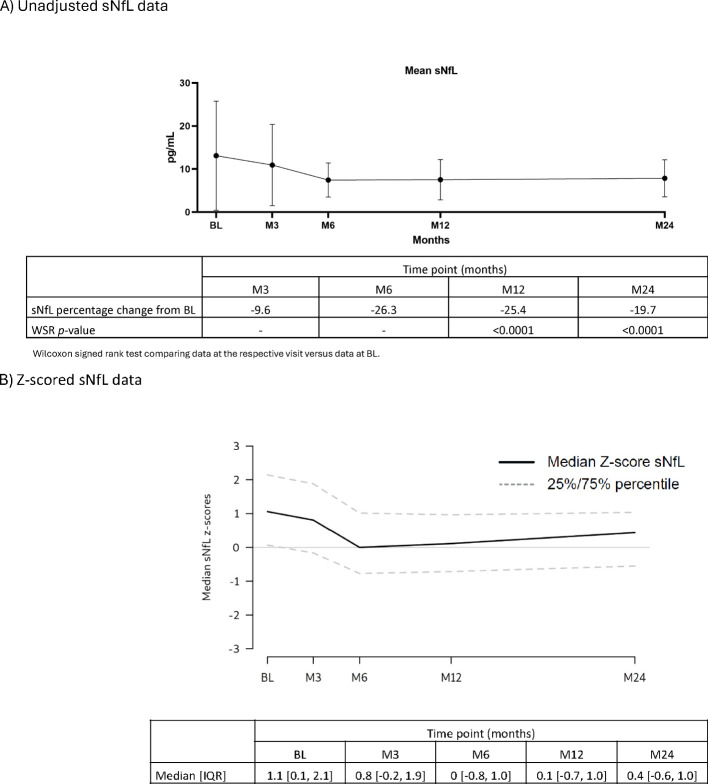
sNfL levels (FAS). **(A)** Unadjusted sNfL data. **(B)** Z-scored sNfL data. BL, baseline; FAS, full analysis set; IQR, interquartile range; M, month; sNfL, serum neurofilament light chain; WSR, Wilcoxon signed rank test.

**Serum proteins:** Throughout the 2-year study, serum IgG and IgM levels for most participants remained within the normal ranges of 5.65–17.65 g/L and 0.40–2.30 g/L, respectively (**Table 2**). For IgG, at BL, M3, M24: >50% – <75% were within the normal range. M6, M12: all participants were within the normal range. M15, M18: >75% of participants were within the normal range. For IgM, at all visits approximately >50% – <75% of participants were within the normal range. Mean sNfL levels at BL were 13.11, and 7.53 and 7.87 ng/L at M12 and M24, respectively. During the study, sNfL levels decreased by 25% at M12 and 23% at M24 (p<0.0001). This reduction was also evident in the normalized Z-scores, at M12 the median sNfL Z-score was 0.1 (a z-score of zero is similar relative to the age- and BMI-matched reference population). sNfL Z-score reduction was sustained at M24 (**Figure 2**).

### Cytokine producing B and T cells

**Figure 3:**
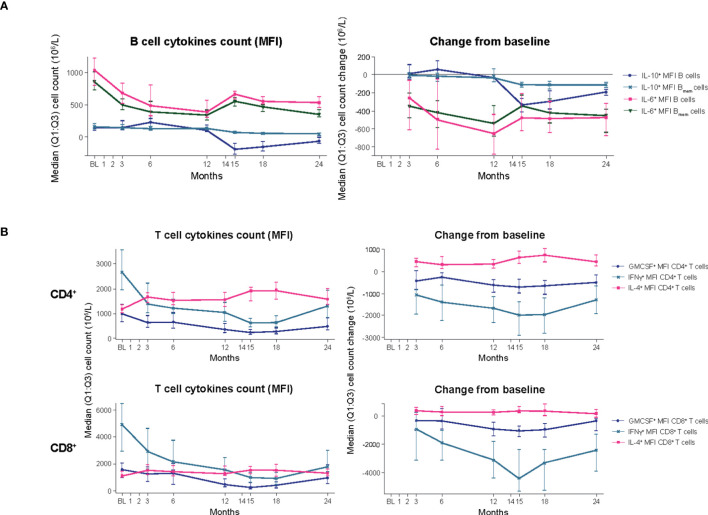
B- and T-cell cytokines (blood biomarker sub-study). **(A)** B-cell cytokines. **(B)** T-cell cytokines. BL, baseline; B_mem_, B memory; CD, cluster of differentiation; GMCSF, granulocyte-macrophage colony-stimulating factor; IFN, interferon; IL, interleukin; M, month; MFI, mean fluorescence intensity; Q1:Q3, quartile range; TNF, tumor necrosis factor.

**B cells:** In the blood biomarker sub-study population, treatment with CladT reduced the level of pro-inflammatory interleukin (IL)-6 producing B_mem_ cells in PBMCs after the first and second yearly treatment courses. IL-6^+^ B_mem_ expression did not reach BL levels at M24 (**Figure 3A**; **Supplementary Table 1**). The reductions for IL-6^+^ B cells and IL-6^+^ B_mem_ cells were most pronounced at the end of the first year of treatment with CladT, with the most pronounced decrease at M12 (median percentage change from BL: -16% and -12%, respectively). There was an increase at M6 in anti-inflammatory cytokine-producing B cell counts (IL-10^+^) following the first course of CladT; however, after the second treatment course, there was a reduction in IL-10^+^-producing B cells. Overall, a slight decrease in IL-10^+^ B cell counts was seen after M12, with a nadir at M15 (-1.6%). IL-10^+^ B_mem_ cells were similarly reduced compared to BL, with a nadir at M15 (-2.5%).

**T cells:** Decreases in cell counts for pro-inflammatory cytokine producing T cells such as granulocyte-macrophage colony-stimulating factor (GMCSF^+^), interferon (IFN)y^+^, and tumor necrosis factor alpha (TNFα^+^) T cells were observed. The reductions in GMCSF^+^ and IFNy^+^ producing CD4^+^ T cell counts were observed from M3 (median percentage change from BL: -3.3% and -3.6%) and continued until M15. For GMCSF^+^ and IFNy^+^ producing CD8^+^ T cells, counts were reduced following the first treatment course, reached a nadir at M15 (-12% and -19%, respectively, **Figure 3B**, **Supplementary Table 1**). These cells did not recover to BL values. Increases or no substantial reduction in cell counts were observed for anti-inflammatory cytokine-producing T cells (IL-4^+^ and IL-10^+^ T cells, respectively). IL-4^+^ CD4^+^ T cell counts increased slightly following the first treatment course (M3, +0.8%) and remained above BL levels to M24 (+1.7%). IL-4^+^ CD8^+^ T cell counts were also slightly increased over BL up to M24 (+0.6%).

### Transcriptomics

**Figure 4:**
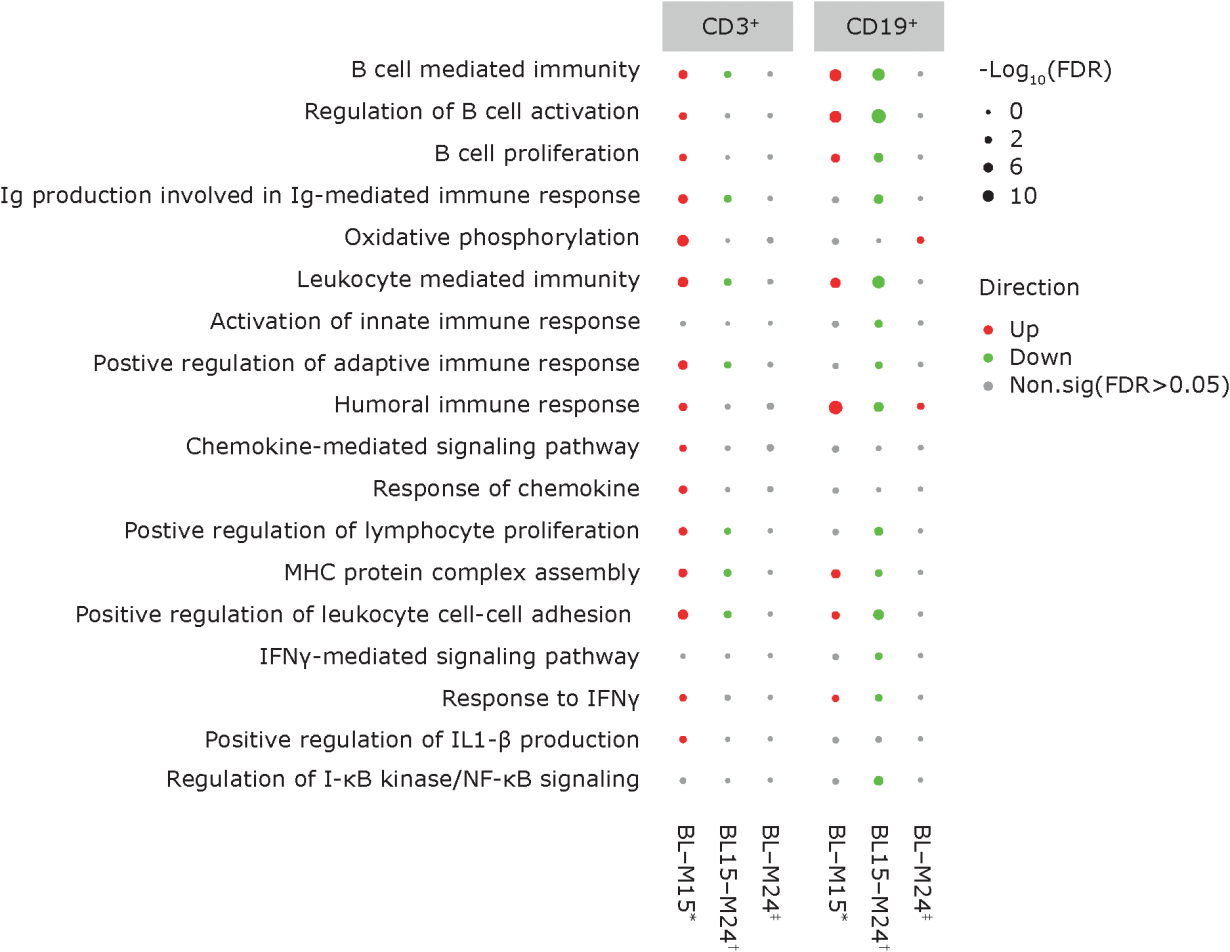
Pathway dynamics during the study. *BL–M15 is considered to be completion of the 1^st^ treatment course as PBMCs were taken before the 2^nd^ treatment course at M12. ^†^M15–M24 is considered to be completion of the full treatment course, and reflects immune reconstitution. ^‡^Describes pathway dynamics during the entire study. BL, baseline; FDR, false discovery rate; IFN, interferon; Ig, immunoglobulin; IL, interleukin; MHC, major histocompatibility complex; M, month; NF, nuclear factor; PBMC, peripheral blood mononuclear cells.

**Figure 5:**
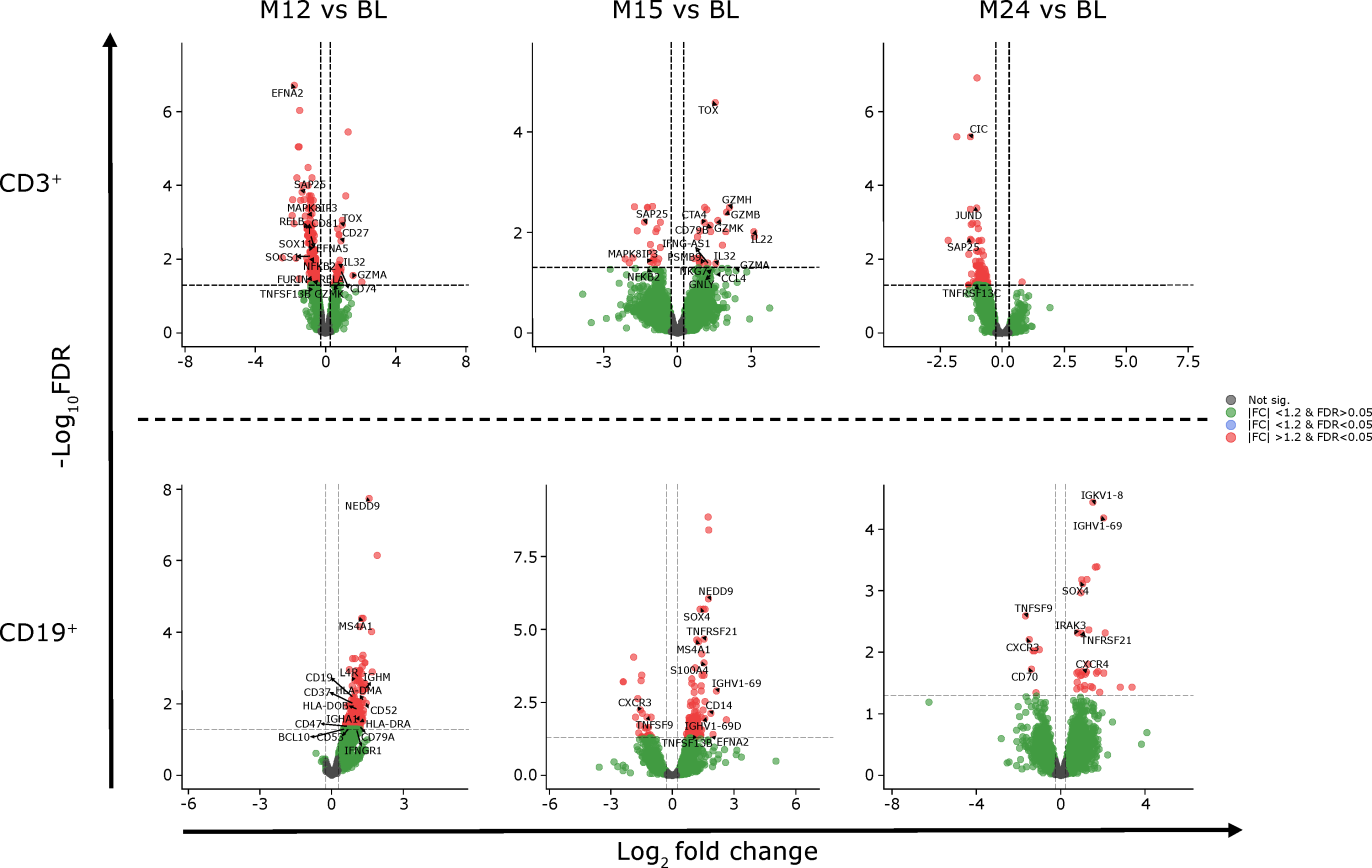
Gene expression of CD3^+^ and CD19^+^ cells at M12, M15 and M24 compared to BL. BL, baseline; FC, fold change; FDR, false discovery rate; M, month.

Differentially expressed genes and the associated pathways were analyzed at each of the reported time points. The most prolific changes were observed after the completion of each yearly CladT treatment course (**Figure 4**). At the end of the 1^st^ treatment course (BL to M12) both B and T cells showed upregulation in humoral immune response genes and pathways (**Figures 4**, **5**). While B cell expression was upregulated (n=229), most T cell transcripts (n=260) were downregulated at this time point (**Supplementary Table 2**). B cells showed an increase in CD19, CD74, CD22, and NF-κB inhibitor (NFκBI) genes, while an increase in granzyme A (GZMA), IL-32 and CD27 genes were seen in T cells, and a decrease in nuclear factor (NF)-κB2. Adaptive and innate immune responses were detected in B cell genes. At the time of completion of the second treatment course (comparing M15 to BL), an increase in B cell immunity and humoral response was seen for both B and T cells. Upregulated genes for CD3^+^ cells included: CDK6, CD27, IL-32, GZM(A, B, H and K), NKG 7, and DNA-binding factor TOX. At this timepoint, transcripts associated with adaptive immune response and regulation of IFNy+ production were upregulated in T cells only. Transcripts upregulated in B cells included SOX4, TNFRSF21, CCl5, and SOD 2. CD19^+^ cells had downregulated chemokine encoding genes at this time point (CXCR3). At the end of the study (BL to M24), upregulation was detected in two pathways only: oxidative phosphorylation and humoral immune response for B cells, otherwise gene expression was like BL. The full list of genes at M12, M15 and M25 compared with BL is presented in **Supplementary Appendix 2**.

### Clinical outcomes

In the FAS, the ARR was 0.11 (95% CI: 0.09, 0.15), with 83% of participants free of qualifying relapses and 91.8% free of 6mCDP during the 24M study period. 92.9% and 96.9% of participants did not have confirmed progression on T25FWT and 9HPT, respectively (**Supplementary Table 3**). Among participants with MRI data (n=265), significant reductions in the annualized CUA count were seen from M2 (**Supplementary Figure 3A**). The maximum mean change was reached at M6 and was maintained until M24. In the total population, 61.7% of participants had no T1 Gd+ lesions and 57.7% had no active T2 lesions (without T1 Gd+) at BL. By M24, this proportion increased to 92.8% and 86.9%, respectively (**Supplementary Figure 3B**). The safety profile observed in this study was generally consistent with that previously reported, with no new safety findings reported during the study. Incidences of study treatment related infections were reported in 55 (20.4%) participants and were either mild or moderate. No severe study treatment related infections were observed (**Supplementary Table 4**).

### Correlations

In participants with both MRI and B cell data, the correlation was found between B_mem_ or B_reg_ cell counts and CUA, T1 Gd+ or active T2 lesions at M3 or M6 was not statistically significant (**Supplementary Figure 4**). Most participants were grouped into the ≥90% category for both correlation analyses. A significant but weak negative correlation was observed between changes in CUA and changes in B_reg_ cells from BL to each M12 and M15 timepoints. No further significant correlations were observed for B_mem_ or other timepoints for B_reg_ cells (**Supplementary Figure 5**). No significant correlations were found between disability status categories and selected immune cell subset ratios, including CD16^bright^CD56^dim^/NK cells and CD16^low^CD56^bright^/NK cells (**Supplementary Figure 2**).

## Discussion

Performing deep immunophenotyping on the entire MAGNIFY-MS cohort at each visit allowed for better understanding of time and extent of immune cell dynamics and its potential effect on clinical outcomes. In this study, we contrasted lymphocyte dynamics with MRI and clinical outcomes, and analysis of transcriptomics. Several observations made in the B, T and NK cell pool point to cladribine’s effect on adaptive immunity and a transient effect on innate immunity. In line with previously reported data (2, 8, 15), we observed a sustained reduction of B_mem_ cells after the first CladT treatment course, and further reduction after the second treatment course (nadir values at M15). B_mem_ cells remained low until the end of the follow-up period at M24. Other studies have demonstrated that a proportion of circulating B_mem_ cells likely harbor latent EBV infection, which in turn drives proliferation of B_mem_ cells (19). We observed a sustained and durable effect of CladT on reduction of these pathogenic cells, which likely results in proportionally reduced numbers of B_mem_ in the CNS thereby blocking ongoing inflammation. We also observed increases in NK cells (both CD56^bright^ and NKp46), which have been shown to limit circulating EBV-infected B cells (20, 21). However, it remains to be elucidated whether increases in NK cells seen in this study are beneficial for controlling EBV infection in MS. In addition, EBV can induce oncogenesis through multiple pathways, one of which is NF-κB (22).

The NF-κB pathway plays a significant role in the pathogenesis of MS. This pathway is crucial for regulating immune responses, inflammation, and cell survival. In MS patients, the NF-κB pathway is often dysregulated, leading to increased activation and contributing to the inflammatory processes that characterize the disease (23, 24). We detected both up- and down-regulation of the genes involved in this pathway. After the first treatment course, genes *NFκB2, RELA* and *RELB* were downregulated in CD3^+^ cells, while NFκBI zeta (*NFκBIZ)* was upregulated in CD19^+^ cells. The decrease of *NFκB2* expression also remained after the completion of the second treatment. The regulation of NF-κB is similar to that seen in fingolimod-treated people who responded to therapy, where NFκBI beta (*NFκBIB*) and *NFκB1* genes are upregulated at 12 months of therapy (25). Unlike fingolimod, CladT does not have a constant immune suppression effect. Instead, during CladT treatment rapid depletion of all lymphocytes including pathogenic B_mem_ cells is observed. This process is followed by repopulation of B_naïve_ and B_trans_ cells, as well as NKp46 cells. Therefore, it is not surprising that up- and down-regulation of specific genes is observed in this important pathway after the first treatment course. Most importantly, we have observed that down-regulation of NFkB2 persisted after the second year of treatment, indicating overall reduction in the inflammatory process. This is also supported by a reduction in pro-inflammatory cytokines.

We detected reconstitution to above BL levels for B_reg_, B_trans_ and B_naïve_ cells. Previously, B_reg_ and B_trans_ cell counts were found to reduce relatively rapidly (-45% and -61%, respectively) and recovered to above baseline levels by M3 (+176 and +28, respectively); increases were demonstrated up to 1 year (15). Here, they remain increased from BL until 24M. B_reg_ cells are known to display suppressive functions toward pro-inflammatory and autoreactive immune responses (26). We noted a substantial increase in B_reg_ counts after the first treatment course (M3), which was maintained after the second treatment course, indicating a sustained beneficial treatment effect of CladT. B_trans_ cell counts were increased starting at M6, with the most substantial increase reported after the second treatment course. Reduced B_trans_ cell levels have been shown to be associated with a high risk of MRI activity in IFN beta-treated patients (27). In addition, the recovery of B_naïve_ cells in the presence of sufficient T cell control may prevent the occurrence of conditions that are needed for secondary autoimmunity (11).

Modification of the adaptive response to infectious agents by high-efficacy treatments for MS has been brought into focus in the recent COVID-19 pandemic, both through increased susceptibility and severity of infections and the potential difficulty of effective vaccination. In our study, IgM and IgG levels remained within reference ranges for most participants. We observed slight reductions in IgM levels, which could be linked to the temporary, but profound reduction in B_naïve_ cell counts, or the change in cytokine production from lymphocytes. In addition, we also observed that genes involved in immunoglobulin production were upregulated in both cell types after completion of the full treatment course (M15). We did not observe reductions in IgG levels. This stability in IgG is of importance to patients, and underpins the modest effect of CladT on plasma cells, as reduced levels of IgG have been linked to an increased risk of infection (28). In CladT-treated patients, immunoglobulin stability may also account for the sustained ability to mount a humoral response to vaccination (29), compared to those treated with other cell depleting or sequestering DMTs which result in reduced humoral responses (30).

Reductions in T cell subset counts were sustained up to the full 2-year treatment course of CladT, with lowest values reached after the second-year dosing at M15 for most subsets, and for CD4^+^ EM, CD4^+^ reg, and CD8^+^ EM at M18. Whilst T cell depletion on its own (31) would not be expected to lead to such significant clinical effects as seen with CladT, reduction in T cell subsets combined with the sustained reduction of B_mem_ cells may explain the durability of CladT treatment effect beyond the dosing period.

Recent observations suggest that the expression of certain cell surface markers by NK cells might be an important indicator of the activation state of the immune system (32). In this study, CD16^low^CD56^bright^ and NKp46 repopulated soon to above BL levels at M6 and M12 respectively, indicating a transient effect of CladT on innate immunity. It is known that activated CD56^bright^ NK cells migrate to the intrathecal compartment in MS and regulate autoreactive T cells via cytotoxicity (33). Additionally, CD56^bright^ NK cells have been proposed to improve outcomes in RMS through immunomodulation (34), and could be a biomarker of treatment response (35). Others reported an association between increased CD56^bright^ NK cells and reduced MRI activity in patients (36). In our study, we did not find any statistically significant correlations between the increase in NK cells and MRI or clinical activity when comparing with the entire cohort of participants. However, we did observe an increase in genes that regulate innate immune response in B cells at M12 and M15. The role of NKp46 activation on NK cells in patients undergoing treatment with CladT remains to be determined; however, the finding that increased NKp46 levels have also been observed in patients after hematopoietic stem cell transplantation is an indicator of reconstitution of the immune system towards a more favorable state (35).

Further evidence that there is a change in the inflammatory state of the immune system after CladT treatment was detected by observation of changes in the number of T and B cells expressing anti- or pro-inflammatory cytokines. Reductions in cells positive for pro-inflammatory cytokines were more pronounced than changes in anti-inflammatory cytokines, with a consistently greater reduction in the second year. These changes in anti-inflammatory and pro-inflammatory cytokine-producing cells give further indication that CladT leads to post-reconstitution qualitative changes, which continue to evolve after completion of the full cumulative dose. Gene expression and pathways analysis also support this observation. For example, CD3^+^ had an upregulation in genes involved in the positive regulation of cytokine production after the full treatment course (M15).

We detected sustained reduction of sNfL to M24, indicating reduced neuroaxonal damage (37) during treatment with CladT. Our results are consistent with previously reported results from real-world observational studies (38, 39). When raw sNfL levels were normalized for age and BMI, it became apparent that they were reduced close to the levels of general population (16) during CladT treatment.

The maximum effect of CladT on MRI activity becomes apparent at 6 months (40) after initiation of treatment, and remained consistently low for the remainder of the 2-year study. These results are similar to those described in the original study (14), but obtained in younger participants judged to be highly active at BL and with a much higher frequency of MRI lesions. The significant reduction in lesion count was maintained up to M24, highlighting the efficacy of CladT in people with highly active RMS. Here, we found no direct correlations between changes in MRI lesion count and B_mem_ or B_reg_ cell counts to 6 months of CladT treatment, nor did we find correlations between clinical and MRI outcomes and ratios of immune cells. We also did not observe broad patient or population-level associations between changes in lymphocytes and CUA lesions from BL to follow-up timepoints. These results are not entirely surprising given similar findings have been published for other DMTs (41–44), strongly suggesting a non-linear and indirect relationship between CUA and B_mem_ rather than a true lack of association. A limitation in our correlation analysis was that the time frame investigated was limited from BL until M6. However, as onset of action was the focus of MAGNIFY-MS, our intention was to determine if the immunological changes caused by CladT could correlate with early MRI outcomes. Our results are supported by previously published data from the MAGNIFY-MS study, where additional analyses including M12, M18 and M24 time points yielded similar results (data not shown) (45). Further analyses on different immune subsets and for specific subgroups of patients over a longer period of time might yield different results. There is further limitation in the transcriptomics analysis. While the pathway and genetic analysis helps further our understanding of how CladT works in MS, the results are restricted by only analyzing CD3^+^ and CD19^+^ cells. Transcriptomics interpretation is also restricted due to low participant numbers. Samples for RNA sequencing were selected at random from 11 participants, with numbers limited by the available budget. Future investigations of more immune cell subsets will help identify the key effects driving disease activity. The immune reconstitution effect seen in MAGNIFY-MS may continue beyond the observed 2-year treatment period. These analyses are ongoing in the 2-year MAGNIFY-MS Extension study (NCT04783935).

## Conclusions

Our findings provide a detailed description of longitudinal peripheral immune effects with subpopulation depletion and repopulation dynamics in a large cohort of RMS patients over the full 2-year CladT treatment course. These results are supported by clinical outcomes and a gene and pathway analysis. Observed patterns of immune cell depletion and reconstitution, support our understanding of CladT action in rebalancing immune system towards a non-pathogenic, immunocompetent state. Particularly the fact that B_mem_ cells and total plasma cells are most strongly influenced and still reduced compared to BL after 2 years, maintenance of IgG and IgM levels within reference ranges, and increases of two populations of NK cells, distinguish CladT from other high-efficacy disease-modifying therapies and continuous immunosuppression approaches. The substantial decrease in sNfL levels indicates reduction of neuroaxonal damage following treatment with CladT. Our findings also highlight the importance of administering the full treatment course of CladT as some observed changes became more prominent in the second year of treatment, consistent with its mechanism of action as an IRT.

## Data Availability

The original contributions presented in the study are included in the article/**Supplementary Material**. Further inquiries can be directed to the corresponding author.
