# Corrigendum: Blood biomarker dynamics in people with relapsing multiple sclerosis treated with cladribine tablets: results of the 2-year MAGNIFY-MS study

**DOI:** 10.3389/fimmu.2025.1571978

**Published:** 2025-03-05

**Authors:** Heinz Wiendl, Frederik Barkhof, Xavier Montalban, Anat Achiron, Tobias Derfuss, Andrew Chan, Suzanne Hodgkinson, Alexandre Prat, Letizia Leocani, Klaus Schmierer, Finn Sellebjerg, Patrick Vermersch, Hulin Jin, Anita Chudecka, Andreas Kloetgen, Dongdong Lin, Lidia Gardner, Nicola De Stefano

**Affiliations:** ^1^ Department of Neurology, Institute of Translational Neurology, University of Münster, Münster, Germany; ^2^ Department of Radiology and Nuclear Medicine, Amsterdam UMC, Vrije Universiteit, Amsterdam, Netherlands; ^3^ Queen Square Institute of Neurology and Centre for Medical Image Computing, University College London, London, United Kingdom; ^4^ Department of Neurology-Neuroimmunology, Centre d’Esclerosi Múltiple de Catalunya (Cemcat), Hospital Universitario Vall d’Hebron, Barcelona, Spain; ^5^ Multiple Sclerosis Center, Sheba Academic Medical Center, Ramat Gan, Israel; ^6^ Faculty of Medicine, Tel-Aviv University, Tel-Aviv, Israel; ^7^ Department of Neurology, University Hospital Basel, Basel, Switzerland; ^8^ Department of Neurology, Inselspital, Bern University Hospital, University of Bern, Bern, Switzerland; ^9^ Ingham Institute for Applied Medical Research, University of New South Wales Medicine and Liverpool Hospital, Sydney, NSW, Australia; ^10^ Department of Neurosciences, Université de Montréal, Montréal, QC, Canada; ^11^ Department of Neurology, University Vita-Salute San Raffaele, Milan, Italy; ^12^ Experimental Neurophysiology Unit, Scientific Institute IRCCS San Raffaele, Milan, Italy; ^13^ Department of Neurorehabilitation Science, Casa di Cura Igea, Milan, Italy; ^14^ The Blizard Institute, Centre for Neuroscience, Surgery and Trauma, Barts and The London School of Medicine and Dentistry, Queen Mary University of London, London, United Kingdom; ^15^ Clinical Board Medicine (Neuroscience), The Royal London Hospital, Barts Health NHS, Trust, London, United Kingdom; ^16^ Danish MS Center, Department of Neurology, Copenhagen University Hospital - Rigshospitalet, Glostrup, Denmark; ^17^ Department of Clinical Medicine, University of Copenhagen, Copenhagen, Denmark; ^18^ Univ. Lille, Inserm U1172 LilNCog, CHU Lille, FHU Precise, Lille, France; ^19^ Clinical Measurement Sciences, Merck Healthcare KGaA, Darmstadt, Germany; ^20^ Clinical Research Services, Cytel Inc., Geneva, Switzerland; ^21^ Clinical Measurement Sciences, EMD Serono Research & Development Institute, Inc., an affiliate of Merck KGaA, Billerica, MA, United States; ^22^ Neurology & Immunology Medical Unit, EMD Serono Research & Development Institute, Inc., an affiliate of Merck KGaA, Billerica, MA, United States; ^23^ Department of Medicine, Surgery and Neuroscience, University of Siena, Siena, Italy

**Keywords:** multiple sclerosis, cladribine tablets, biomarkers, transcriptomics, immunophenotyping, immune reconstitution therapy

In the published article, there was an error in **Figure 5** as published. In the figure, the legend for the blue and red dots incorrectly read as “|FC| <1.2 & FDR>0.05”. The legend for blue should read “|FC| <1.2 & FDR<0.05” and for red should read “|FC| >1.2 & FDR<0.05”. The corrected **Figure 5** and its caption “Gene expression of CD3+ and CD19+ cells at M12, M15 and M24 compared to BL. BL, baseline; FC, fold change; FDR, false discovery rate; M, month.” appear below.

**Figure 5:**
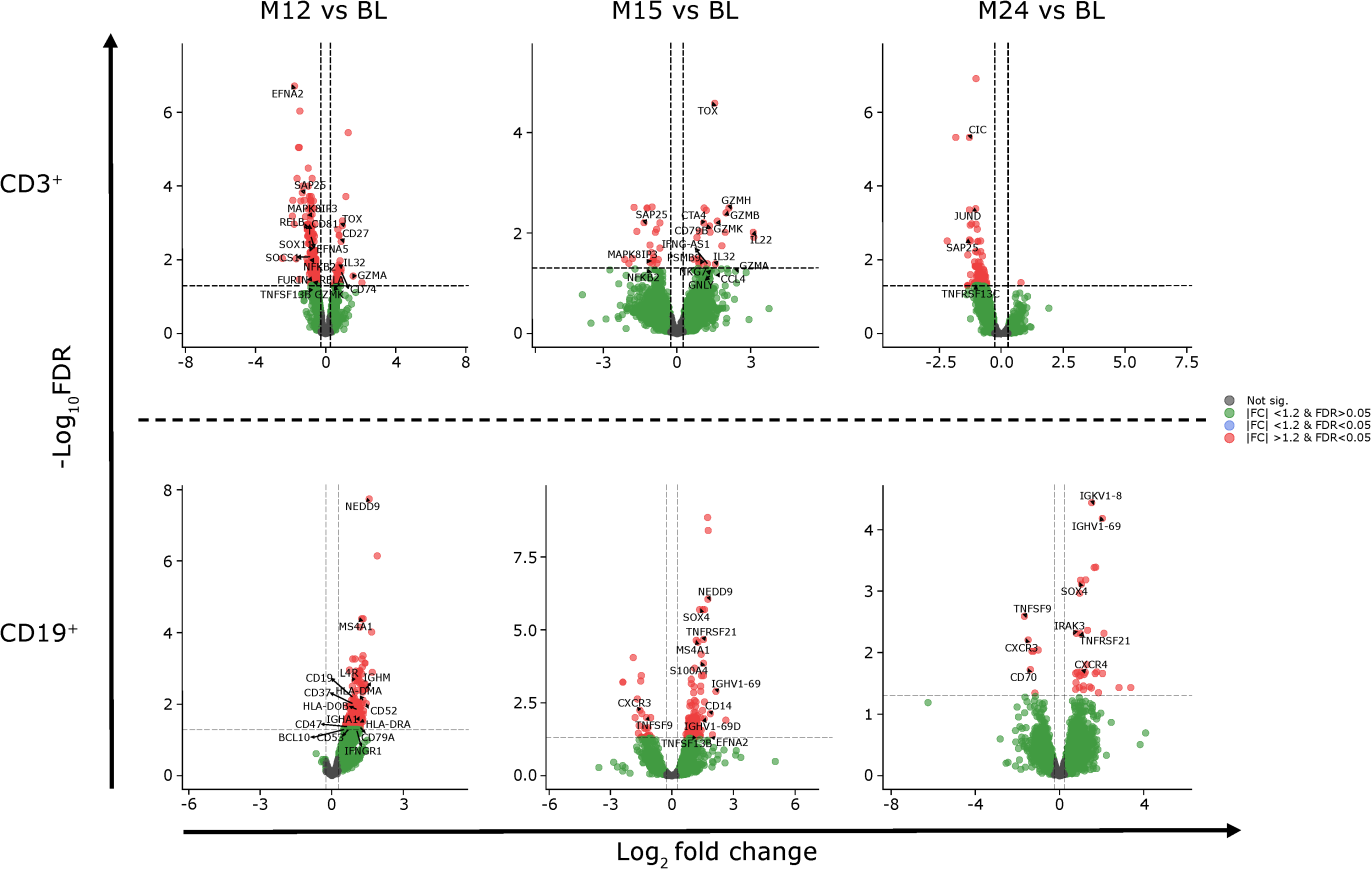
Gene expression of CD3^+^ and CD19^+^ cells at M12, M15 and M24 compared to BL. BL, baseline; FC, fold change; FDR, false discovery rate; M, month.

The authors apologize for this error and state that this does not change the scientific conclusions of the article in any way. The original article has been updated.

