# Supplementary material for: Blood biomarker dynamics in people with relapsing multiple sclerosis treated with cladribine tablets: results of the 2-year MAGNIFY-MS study
Source: Front Immunol. 2025 Feb 3;16:1512189. doi: 10.3389/fimmu.2025.1512189 (PMC11830603; doi:10.3389/fimmu.2025.1512189)
Supplement: Supplementary file 1 [file DataSheet1.docx]

# Supplementary Appendix

##
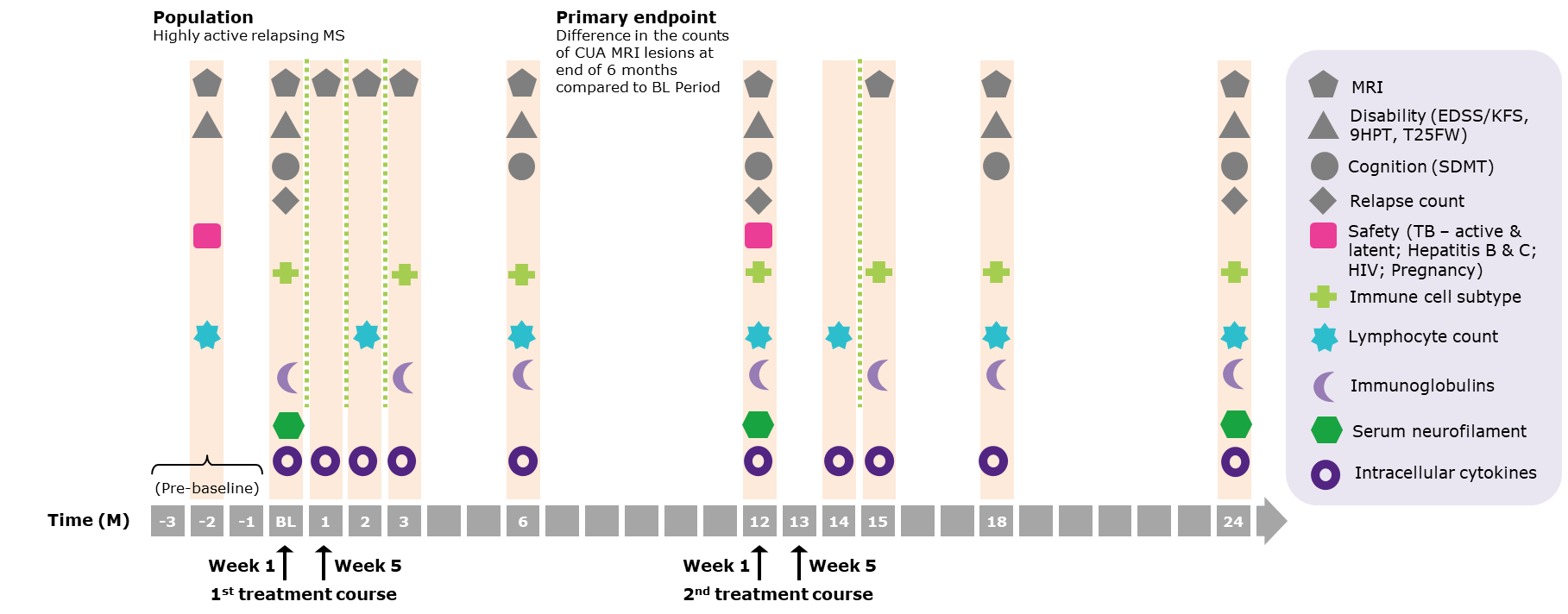
Supplementary Figure 1. Study Design

CladT were administered as two 5-day treatment courses at the beginning of each year: at the beginning of M0 and M1, and at the beginning of M12 and 13. After the baseline visit (visit 1, M0), all visits have a window of ±7 days. Participants enrolled in the blood biomarker sub-study required blood collection for B cell and serum protein analyses at additional time points to those in the core study (M1, 2, and 14).

9HPT, 9-Hole Peg Test; CladT, cladribine tablets; CUA, combined unique active; EDSS, Expanded Disability Status Scale; HIV, human immunodeficiency virus; KFS, Kurtzke Functional System; M, month; MRI, magnetic resonance imaging; SDMT, Symbol Digit Modalities Test; T25FW, Timed 25-Foot Walk; TB, tuberculosis

##
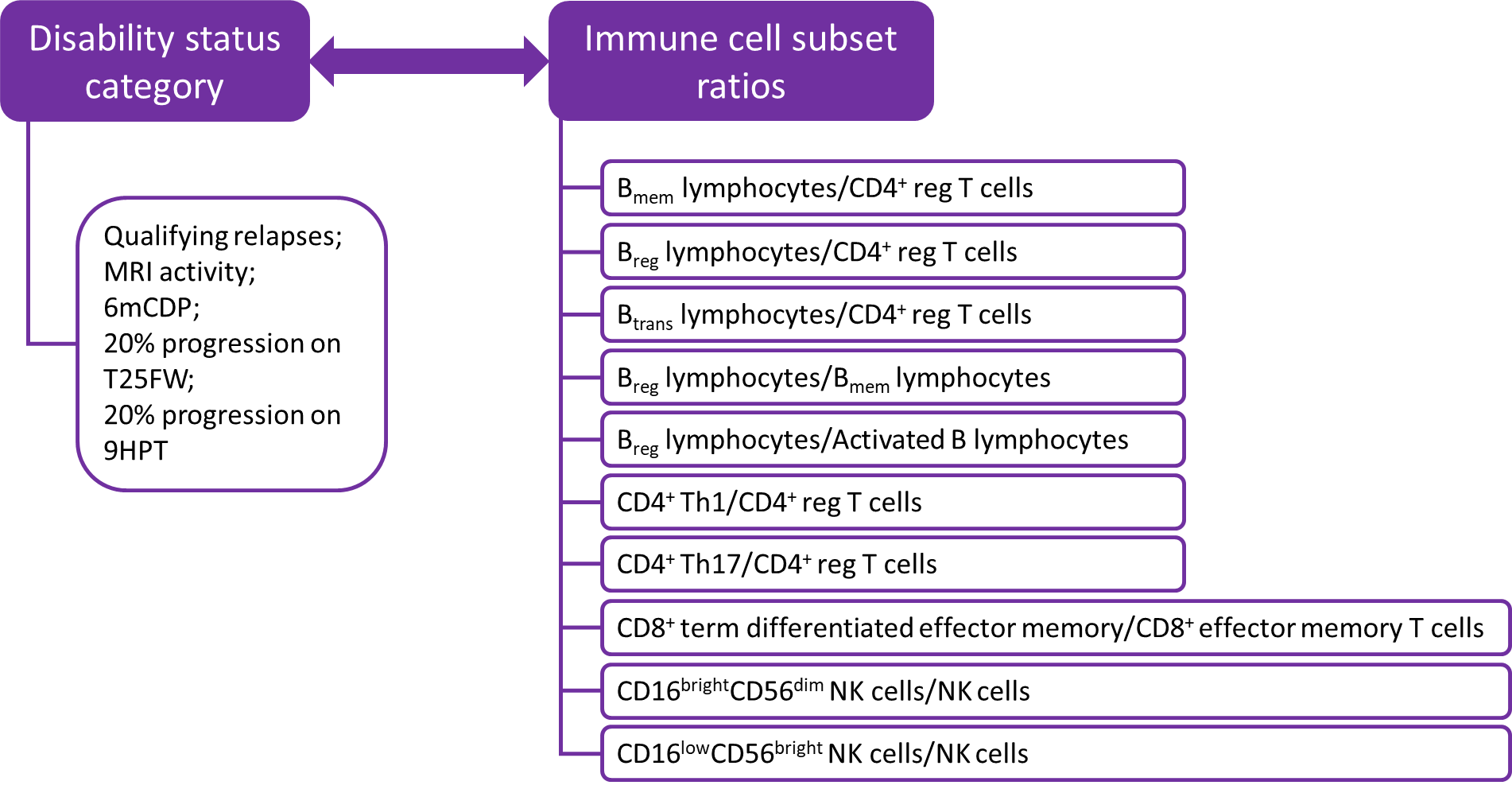
Supplementary Figure 2: Associations Between Immune Cell Ratios and Disability Status Categories

No associations were found between disability status categories and selected immune cell subset ratios.

6mCDP, 6-month confirmed disability progression; 9HPT, 9-Hole Peg Test; B_mem_, B memory; B_reg_, B regulatory; B_trans_, B transitional; CD, cluster of differentiation; EM, effector memory; NK, natural killer; reg, regulatory; T25FW, timed 25-Foot Walk; Th, T helper cell type

## Supplementary Figure 3: MRI Activity (FAS)

1.
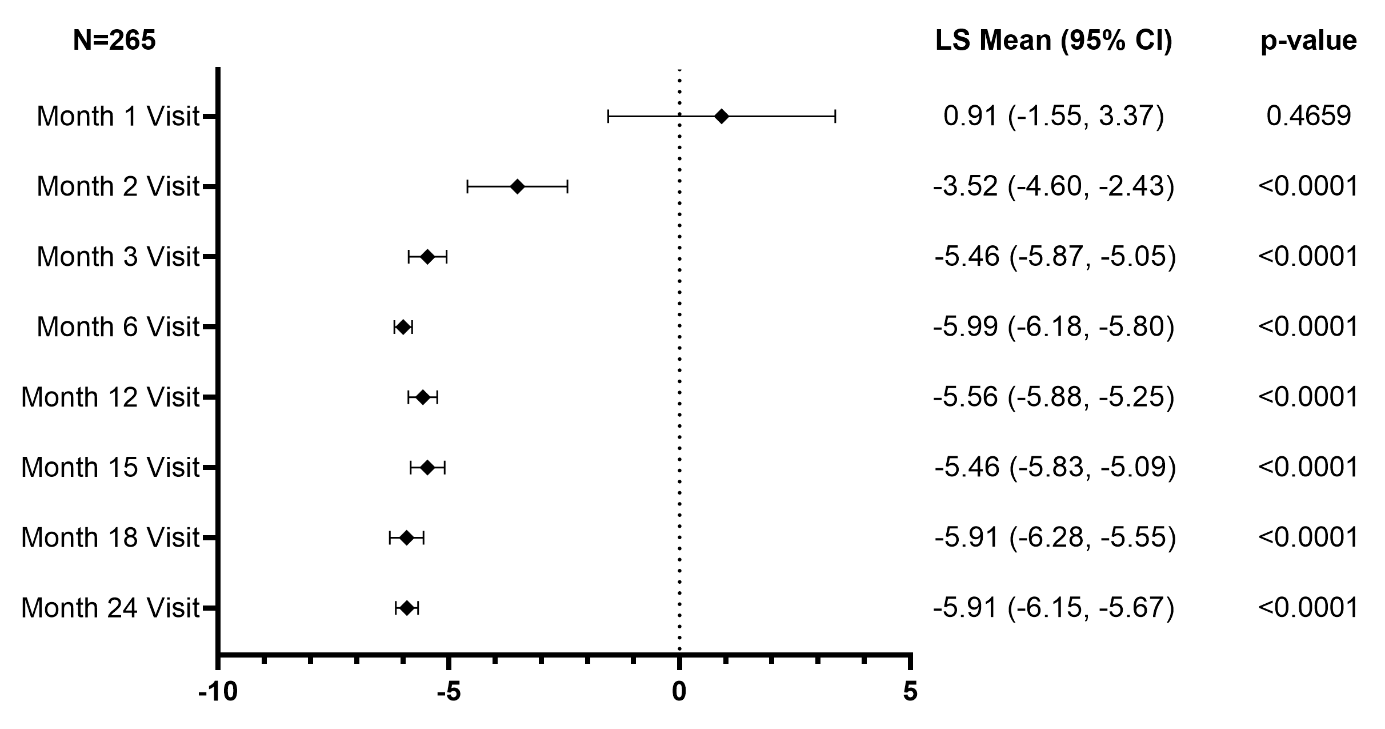
Annualized CUA Lesion Count – Change from Baseline Visit to Post-Baseline Visits
2. T1 Gd+ and T2 Active Lesions Over Time (%)

CI, confidence interval; CUA, combined unique active; FAS, full analysis set; Gd+, gadolinium-enhancing; LS, least square; MRI, magnetic resonance imaging

## Supplementary Figure 4. Correlation of B-cells with MRI findings (FAS)


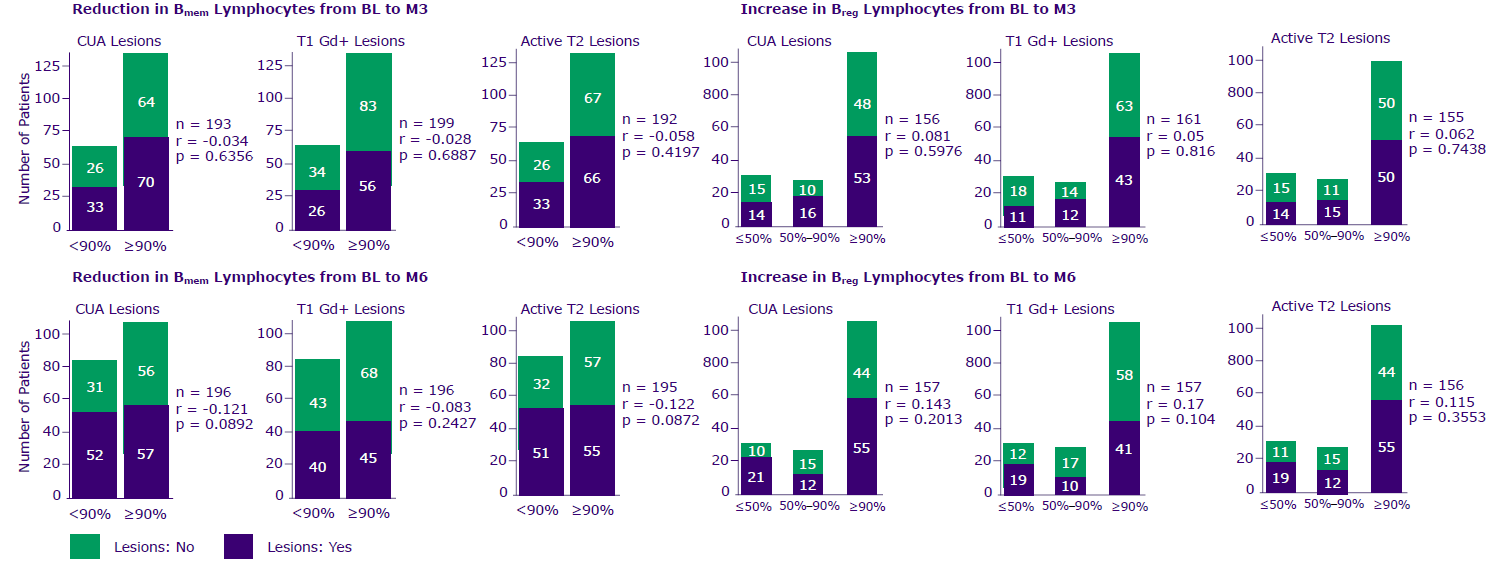


Cramer's V is a measure of association between two nominal variables. In absolute values, it ranges from 0 to 1, where 0 indicates no association between the two variables and 1 indicates a perfect association between the two variables. p < 0.05 is considered significant.

BL, baseline; B_mem,_B memory; B_reg_, B regulatory; CUA, combined unique active; FAS, full analysis set; Gd+, gadolinium-enhancing; M, month; MRI, magnetic resonance imaging; n, number of participants with both B-cell and MRI assessments; r, Cramer’s V correlation coefficient

## Supplementary Figure 5. Correlation of B-cells with CUA lesions to M24 (FAS)


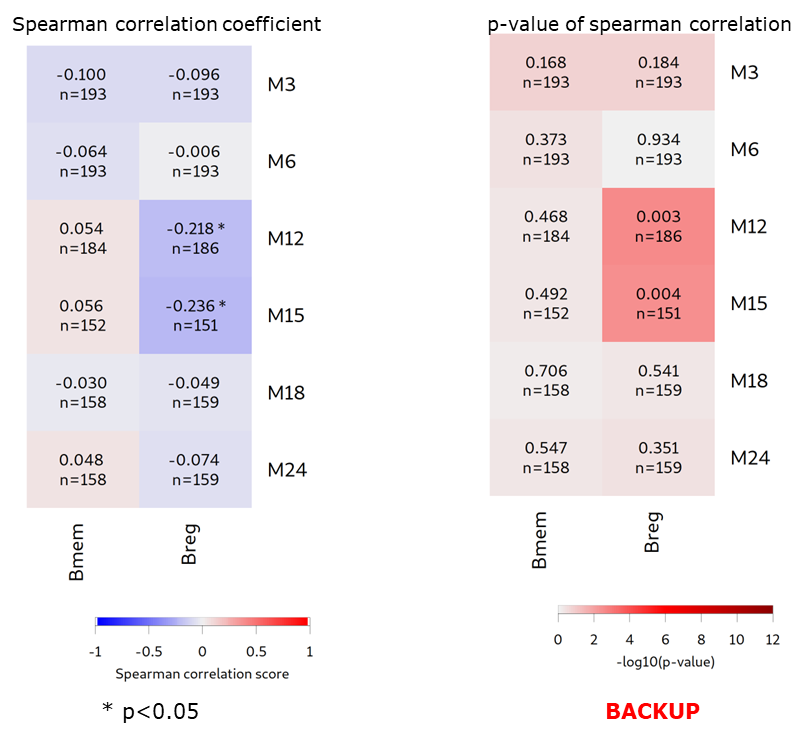


Bmem, B memory; Breg, B regulatory; CUA, combined unique active; FAS, full analysis set; M, month

## Supplementary Table 1: B- and T-cell cytokines (blood biomarker sub-study)

|  | **Intracellular cytokines: Median absolute change (%) from baseline** | | | | | |
| --- | --- | --- | --- | --- | --- | --- |
| MFI | **M3** | **M6** | **M12** | **M15** | **M18** | **M24** |
| B-cell cytokines | | | | | | |
| IL-10^+^ B cells | 1.9 | 1.1 | -0.9 | -1.6 | -1.6 | -0.8 |
| IL-10^+^ B_mem_ cells | -0.9 | -1.4 | -1.5 | -2.5 | -1.5 | -1.4 |
| IL-6^+^ B cells | -7.8 | -9.0 | -16 | -15 | -16 | -13 |
| IL-6^+^ B_mem_ cells | -8.7 | -9.5 | -12 | -11 | -12 | -11 |
| T-cell cytokines | | | | | | |
| GMCSF^+^ CD4^+^ T cells | -3.3 | -1.6 | -5.7 | -6.7 | -5.4 | -3.9 |
| IFNγ^+^ CD4^+^ T cells | -3.6 | -4.1 | -6.1 | -9.2 | -7.0 | -5.2 |
| TNFα^+^ CD4^+^ T cells | -12 | -16 | -26 | -46 | -48 | -29 |
| IL-4^+^ CD4^+^ T cells | 0.8 | 0.8 | 0.6 | 0.9 | 2.3 | 1.7 |
| IL-10^+^ CD4^+^ T cells | 1.8 | 0.5 | -0.3 | -0.9 | -1.2 | -0.9 |
| GMCSF^+^ CD8^+^ T cells | -3.8 | -2.8 | -11 | -12 | -9.6 | -4.7 |
| IFNγ^+^ CD8^+^ T cells | -0.3 | -3.1 | -15 | -19 | -15 | -12 |
| TNFα^+^ CD8^+^ T cells | -14 | -15 | -28 | -45 | -49 | -28 |
| IL-4^+^ CD8^+^ T cells | 0.4 | 0.7 | 0.2 | 0.5 | 1.2 | 0.6 |
| IL-10^+^ CD8^+^ T cells | -0.01 | -0.2 | -0.7 | -1.2 | -1.0 | -0.9 |

In the table, light green shading signifies value above Baseline level; dark green shading, highest value above Baseline level; red shading, nadir value.

B_mem_, B memory; CD, cluster of differentiation; GMCSF, granulocyte-macrophage colony-stimulating factor; IFN, interferon; IL, interleukin; M, month; MFI, mean fluorescence intensity; TNF, tumor necrosis factor

## Supplementary Table 2. Summary of Differentially Expressed Genes

| **Comparison** | **Up-regulated genes, n** | **Down-regulated genes, n** |
| --- | --- | --- |
| M3 vs BL (CD3^+^) | 1 (CD36) | 13 |
| M3 vs BL (CD19^+^) | 1 (IGLV3-27) | 2: LBHD1: JAM3 |
| M12 vs BL (CD3^+^) | 41 | 247 |
| M12 vs BL (CD19^+^) | 399 | 0 |
| M15 vs M3 (CD3^+^) | 0 | 0 |
| M15 vs M3 (CD19^+^) | 0 | 0 |
| M15 vs M12 (CD3^+^) | 9 | 0 |
| M15 vs M12 (CD19^+^) | 26 | 10 |
| M15 vs BL (CD3^+^) | 51 | 34 |
| M15 vs BL (CD19^+^) | 101 | 19 |
| M24 vs M12 (CD3^+^) | 3 | 0 |
| M24 vs M12 (CD19^+^) | 1 | 9 |
| M24 vs BL (CD3^+^) | 1 (FAM13A) | 44 |
| M24 vs BL (CD19^+^) | 24 | 15 |
| M24 vs M15 (CD3^+^) | 0 | 1 (S100A9) |
| M24 vs M15 (CD19^+^) | 107 | 195 |

Fold Change>1.2; False Discovery Rate<0.05

BL, baseline; M, month

## Supplementary Table 3. Disability Status During Treatment Period (FAS)

|  | Total  (N=270) |
| --- | --- |
| Annualized relapse rate (95% CI) | 0.11 (0.09, 0.15) |
| No qualifying relapse, n (%) | 192 (71.1) |
| Unknown status | 32 (11.9) |
| No 6-month confirmed disability progression, n (%) | 236 (87.4) |
| Unknown status | 13 (4.8) |
| No 20% confirmed T25FW progression during treatment, n (%) | 234 (86.7) |
| Unknown status | 18 (6.7) |
| No 20% confirmed 9HPT during treatment, n (%) | 247 (91.5) |
| Unknown status | 15 (5.6) |

9HPT, 9-hole peg test; CI, confidence interval; EDSS, Expanded Disability Status Scale; FAS, full analysis set; T25FW, timed 25-foot walk

## Supplementary Table 4. Summary of TEAEs and infections (FAS)

| n (%) | Total  (N=270) |
| --- | --- |
| Any TEAE^(a)^ | 227 (84.1) |
| Mild | 114 (42.2) |
| Moderate | 103 (38.1) |
| Severe | 10 (3.7) |
| Any study treatment-related TEAE^(a)^ | 122 (45.2) |
| Mild | 71 (26.3) |
| Moderate | 47 (17.4) |
| Severe | 4 (1.5) |
| Any serious TEAE | 14 (5.2) |
| Any study treatment-related serious TEAE | 0 (0) |
| Any TEAE leading to temporary discontinuation of study treatment | 4 (1.5) |
| Any TEAE leading to permanent discontinuation of study treat | 1 (0.4) |
| Any TEAE leading to death | 0 (0) |
| Any infection and infestation | 171 (63.3) |
| Mild | 112 (41.5) |
| Moderate | 58 (21.5) |
| Severe | 1 (0.4) |
| Any study treatment related infection and infestation | 55 (20.4) |
| Mild | 34 (12.6) |
| Moderate | 21 (7.8) |
| Severe | 0 (0) |
| Any study treatment related infection and infestation in ≥5 participants |  |
| Nasopharyngitis | 14 (5.2) |
| Urinary tract infection | 10 (3.7) |
| Oral herpes | 9 (3.3) |
| Upper respiratory tract infection | 7 (2.6) |

a. Worst severity per participant is reported.

FAS, full analysis set; TEAE, treatment-emergent adverse event

## Supplementary Appendix

### Supplementary Appendix 1. Additional Methodology

RNA sequencing: Raw fastq data were aligned to the EnsemblGRCh38 reference genome by STAR software^1^ for quality control. Samples with low read mapping ratio (<60%) were excluded. Next, quantification of read data was performed using Kallisto^2^ for analysis. Differential gene expression analysis was implemented between time points using the DESeq2 package.^3^ Genes with a fold change >1.2 and false discovery rate (FDR) <0.05 were considered significant. Gene set enrichment analysis using the function gseGO in the R package clusterProfiler was applied to examine the enrichment of differentially expressed genes in the pathways from Gene Ontology database.^4,5^ Pathways enrichment with FDR threshold <0.05 were considered significant.

Clinical measures definitions:

6-Months Confirmed Disability Progression (6mCDP): defined as sustained increase in EDSS score of at least 1.5 points if the baseline EDSS score was 0, or at least 1 point if baseline EDSS score was between 0.5 and 4.5 inclusively, or at least 0.5 point if the Baseline EDSS score was at least 5, that occurs over a 6-month time period. The increase was defined as sustained when it occurred on two post-BL Visits, which were at least 166 days apart and no observations at any other Visit (including unscheduled Visits) in between are less than the defined increase.

Progression on T25FW or 9HPT: was defined as 20% confirmed progression by an increase of at least 20% from BL that is sustained over a 6-month time period.

Correlations:

The change in immune cell subsets was predefined as: reduction of **B_mem_** cells (<90%, ≥90% percentage decrease from BL); increase of **B_reg_** cells (≤50%, 50%–<90%, ≥90% percentage increase from BL). Presence of MRI lesions was defined as: CUA, T1 Gd+, or new or enlarging T2 lesion count >0.

**References:**

1. Dobin A, Davis CA, Schlesinger F, et al. STAR: ultrafast universal RNA-seq aligner. Bioinformatics. 2012;29(1):15-21. doi:10.1093/bioinformatics/bts635

2. Bray NL, Pimentel H, Melsted P, Pachter L. Near-optimal probabilistic RNA-seq quantification. Nature Biotechnology. 2016/05/01 2016;34(5):525-527. doi:10.1038/nbt.3519

3. Love MI, Huber W, Anders S. Moderated estimation of fold change and dispersion for RNA-seq data with DESeq2. Genome Biology. 2014/12/05 2014;15(12):550. doi:10.1186/s13059-014-0550-8

4. Yu G, Wang LG, Han Y, He QY. clusterProfiler: an R package for comparing biological themes among gene clusters. Omics: a journal of integrative biology. May 2012;16(5):284-7. doi:10.1089/omi.2011.0118

5. Ashburner M, Ball CA, Blake JA, et al. Gene ontology: tool for the unification of biology. The Gene Ontology Consortium. Nature genetics. May 2000;25(1):25-9. doi:10.1038/75556

### Supplementary Appendix 2. List of Genes at M12, M15 and M24 compared with Baseline
